# Quantified multidimensional public sentiment characteristics on social media for public opinion management: Evidence from the COVID-19 pandemic

**DOI:** 10.3389/fpubh.2023.1097796

**Published:** 2023-03-16

**Authors:** Ning Ma, Guang Yu, Xin Jin, Xiaoqian Zhu

**Affiliations:** ^1^School of Management, Harbin Institute of Technology, Harbin, China; ^2^School of Humanities, Social Sciences and Law, Harbin Institute of Technology, Harbin, China

**Keywords:** public sentiments, sentiments characteristics, audience sentiments, social media, pretraining model, public opinion

## Abstract

**Background:**

Public sentiments arising from public opinion communication pose a serious psychological risk to public and interfere the communication of nonpharmacological intervention information during the COVID-19 pandemic. Problems caused by public sentiments need to be timely addressed and resolved to support public opinion management.

**Objective:**

This study aims to investigate the quantified multidimensional public sentiments characteristics for helping solve the public sentiments issues and strengthen public opinion management.

**Methods:**

This study collected the user interaction data from the Weibo platform, including 73,604 Weibo posts and 1,811,703 Weibo comments. Deep learning based on pretraining model, topics clustering and correlation analysis were used to conduct quantitative analysis on time series characteristics, content-based characteristics and audience response characteristics of public sentiments in public opinion during the pandemic.

**Results:**

The research findings were as follows: first, public sentiments erupted after priming, and the time series of public sentiments had window periods. Second, public sentiments were related to public discussion topics. The more negative the audience sentiments were, the more deeply the public participated in public discussions. Third, audience sentiments were independent of Weibo posts and user attributes, the steering role of opinion leaders was invalid in changing audience sentiments.

**Discussion:**

Since the COVID-19 pandemic, there has been an increasing demand for public opinion management on social media. Our study on the quantified multidimensional public sentiments characteristics is one of the methodological contributions to reinforce public opinion management from a practical perspective.

## 1. Introduction

With the prevalence of COVID-19 (Corona Virus Disease 2019) in the world (1), researchers had paid attention to issues related to information dissemination about COVID-19, including the discussion on COVID-19 vaccine (2), related false health information (3) and misinformation (4). The spread of COVID-19 was accompanied by public opinion communication including not only rumors or misinformation but also pandemic dynamics, socioeconomic issues, public opinion, etc. Thus, in addition to fighting the pandemic (5), it appears necessary for governments to prevent the spread of “information epidemic”, and complete public opinion management.

This pandemic has caused both physical and psychological trauma among the public in various countries (6, 7). At the same time, public opinion communication have caused the emergence of a large number of public sentiments, and affected the psychological state of public (8). Public opinion events such as “Shuanghuanglian inhibits COVID-19”, “Shouguang vegetable”, etc., which were misreported news or misinformed health information, caused the growth of psychological epidemic and sentiments among the public (9). Public comments with spontaneous participation of the public for specific issues on social media will lead to the spread of some public sentiments (10), and this problem became worse during the COVID-19 pandemic (11). As a result, while the efforts to combat the pandemic were seriously negatively impacted, the public was simultaneously exposed to threats of massive negative public sentiments due to the pandemic. Public opinion crises revealed in various events have attracted the attention of managers, and various service measures have been gradually implemented (12). However, no matter in the epidemic outbreak stage or in the current normalized management stage, there are still many public opinions that have not been managed in a timely and accurate manner, and public sentiments problems have been exposed repeatedly in social media.

Since the public discovers events festering on social media earlier and disseminates these ideas through the Internet, the spread of public anxiety and panic occurs before managers have an opportunity to react. It is necessary to establish a method to effectively monitor and manage public opinion crises to prevent the spread of negative sentiments caused by public opinion communication (13). Governments should apply social media interaction data to analyze public sentiments using artificial intelligence technology to provide a scientific basis for formulating effective communication strategies to handle public opinion crisis and stabilize general confidence and cohesion in controlling the pandemic. The objective of this research is to answer two questions: (1) What are the multidimensional characteristics of public sentiments in public opinion during the pandemic? and (2) How to quantify and analyze multidimensional public sentiments characteristics? The results provided an effective means and decision basis for governments to establish reasonable interventions to enhance public opinion management during the pandemic.

## 2. Literature review

### 2.1. Investigating public opinions through social media

Existing measures regarding emotion and cognition are usually administered through retrospective questionnaires such as the satisfaction with life scale (14), Oxford happiness questionnaire (15), and Likert-type attitude scale (16). However, due to the sudden nature of COVID-19, we were unable to measure public sentiments and perceptions in advance using traditional questionnaire methods.

Social media not only provides a platform for the general public to share their lives but can also be used to test public opinions (17). The “big data” generated by the public's daily activities on social media has been intensively studied and widely used in recent years (18). This source of information is, in a sense, more effective than public opinion gathered through the traditional method of questionnaires (19). Accordingly, an analysis of public opinion during COVID-19 in conjunction with information gathered from social media is essential to improve the emergency response, disseminate proper information, increase emotional awareness, and surveil infodemics (3).

Social media plays a crucial role in people's perceptions, decision-making, and behaviors regarding information about the outbreak (20). However, since most information on social media is user-generated, potentially subjective or inaccurate messages often lead to misinformation or conspiracy theories (21). Therefore, governments must disseminate accurate and timely information to the public regarding these threats. Extracting valuable data from social media to provide governments with effective information is crucial when dealing with significant social issues with great impacts such as COVID-19 (22). Social media plays an essential role in providing accurate information during COVID-19 (23). Simply sharing updates on outbreak developments and policies may no longer be sufficient to draw public attention to this information. Governments need to adopt a more compassionate approach to communication to address national and global public concerns (24). Governments should pay close attention to anomalies in social media data to improve their acute perception to public sentiments and public opinion.

### 2.2. Public sentiments analysis in social media during COVID-19

Uncertainty caused by COVID-19 may affect public perception assessments and trigger more negative sentiments (25). When faced with a threat like COVID-19, people tend to strictly adhere to social norms for self-protection and develop avoidance behaviors and negative cognitive assessments (26, 27). According to the interactive ritual chain theory, people in crisis situations are more inclined to seek emotional support but inevitably have different positions (28). Hence, the study of public sentiments has become an important part of public opinion research.

Researchers have made extensive achievements in exploring public sentiments with the method of sentiments analysis. Some studies not only found that the messages of government handling pandemic will have a positive impact on public sentiments, but also found the importance of the emotional value of Weibo posts (29). Positive and negative emotions predicted increased social media use whereas the interaction of negative emotions predicted social media use during the COVID-19 pandemic (30). However, some people also found the phenomenon of exacerbate anger in social media (31). There were an increase in the sensitivity of negative sentiments to social risks and a decrease in positive sentiments and life satisfaction (32). There are four general categories of sentiment analysis applications for massive amounts of social media data at present (33). Since psychological changes due to public opinion communication can be directly reflected in sentiments (34), it is critical to understand and monitor public sentiments to provide basis for public opinion management.

### 2.3. Public sentiments and public topics during COVID-19

Emotional states can be communicated to others through social media (35). Some individuals were more susceptible to social contagion effects and more likely to experience anxiety in response to pandemic threat (36), their discussion on social media conveyed this anxiety at any time. Due to COVID-19, many employees were strongly encouraged or forced to work from home and developed negative attitudes toward remote work (37). When the pandemic hit, social media use was beneficial to people to gain information, emotional support. However, some psychological issues caused by the overuse of social media were also discussed (38). Public also paid attention to topics about vaccines, and the relevant investigations of public sentiments and public topics were also very substantial (39, 40). Some people were anxious about being infected by the virus, this issue also attracted the attention of scholars to explore new coping strategies to reduce anxiety (41). Therefore, to prevent and mitigate the negative effects of COVID-19, the government must implement the necessary interventions to response to public sentiments crisis (42).

Rapid advances in machine learning and text-mining techniques have facilitated the analysis of social media data to understand human behavior, public reactions, potential courses of action, and public opinion in emergency situations (43). Analysis of COVID-19 social media data allowed for topic extraction and construction of classification models and subsequent development of accurate responses designed based on public needs (44). Some scholars have analyzed sentiment tendencies and trends of popular topics related to COVID-19, and performed visual clustering analyses of the content of popular topics (17). Researchers also used deep learning algorithms such as LSTM to classify public sentiments and analyze public topics (45). The study on public sentiments also adopted the method of BERT models combined with topic analysis, and the researchers explored the classification of public sentiments and topics discussed by public (46). Therefore, the measurement of attitudes and sentiments of public comments can portray the changes in online public opinion in a more comprehensive way (47). The recent research also carried out descriptive statistical analysis on the publishing mode, keywords use and sentiments of Weibo posts published by different genders on social media platforms during the COVID-19 pandemic (48).

### 2.4. Multidimensional public sentiments characteristics for public opinion communication

Previously, scholars have conducted considerable exploratory work using social media data to investigate public online sentiments in major outbreak events and have proposed many relevant research methods that provide a reference for the development of relevant government strategies that have laid the necessary foundation for our research (3, 49). However, to reveal the impacts patterns of public opinion communication in terms of public sentiments, we still need to solve the quantization problem of multidimensional public sentiments characteristics.

Few studies conducted independent discussions on posts and comments on platforms like Weibo and Twitter, and most studies explored the scientific issues contained in the posts (48). The public sentiments in this study were divided into two parts for characteristics quantification: The public sentiments of Weibo posts can also be called posts sentiments, and the public sentiments of Weibo comments can also be called audience sentiments. These comments could be regarded as the content published by the audience of the posts. The characteristics of posts sentiments and audience sentiments were discussed separately to find the similarities and differences.

The current research on public sentiments were able to describe the macro trend (50), but the quantification of time series characteristics of public sentiments was not detailed enough. Whether there was a certain pattern in the time series of public sentiments, and how to describe the specific pattern were proved to be the first questions in this study. Here, I proposed the following questions about public sentiments time series characteristics in this study:

*RQ1*: When did the public sentiments explode? Did the public opinion erupt at the beginning or after the priming of some key points in the public discussion?

*RQ2*: Was there a certain pattern in the public sentiments time series? If there was a certain pattern, was the pattern displayed in posts sentiments consistent with that in audience sentiments?

Most studies separated public sentiments from public topics, and most of the combined studies simply analyzed the descriptive differences of public topics based on public sentiments. The relationship and the mining of the features of public sentiments in content were rarely involved. However, previous studies found that if there was irrational verbal aggression, not only public sentiments were impacted (51), irrational verbal aggression also changed public attitudes and behaviors and caused the increase in aggressive speech use in online discussions (52, 53). Such discovery required us to pay attention to the relationship between public sentiments and public topics, and quantify the content-based characteristics of public sentiments, the above turned into the third question of this study:

*RQ3*: What kinds of relevance existed in public sentiments and public topics and how to quantify the relevance? What were the similarities and differences between the performance of the relevance in posts sentiments and audience sentiments?

Public venting is an easy way to let off steam, especially on social media, which is an anonymous textual discussion environment (54, 55). In the context of the pandemic, the public's fragile psychological state was disrupted by public opinion (9). Exploring the relationship between public sentiments and the information they were exposed to when public discussed in such environment provided the inspiration for exploring the relationship between posts sentiments and their audience sentiments. Audience sentiments are the sentiments that the audience of the post responds to. Therefore, audience sentiments are the most direct reflection of the public on the information and posts they contact. The fourth question of this study was how to mine the audience response characteristics shown by audience sentiments:

*RQ4*: What factors affected the audience sentiments? What role did the audience response characteristics shown by the audience sentiments play in public opinion management?

**Figure 1:**
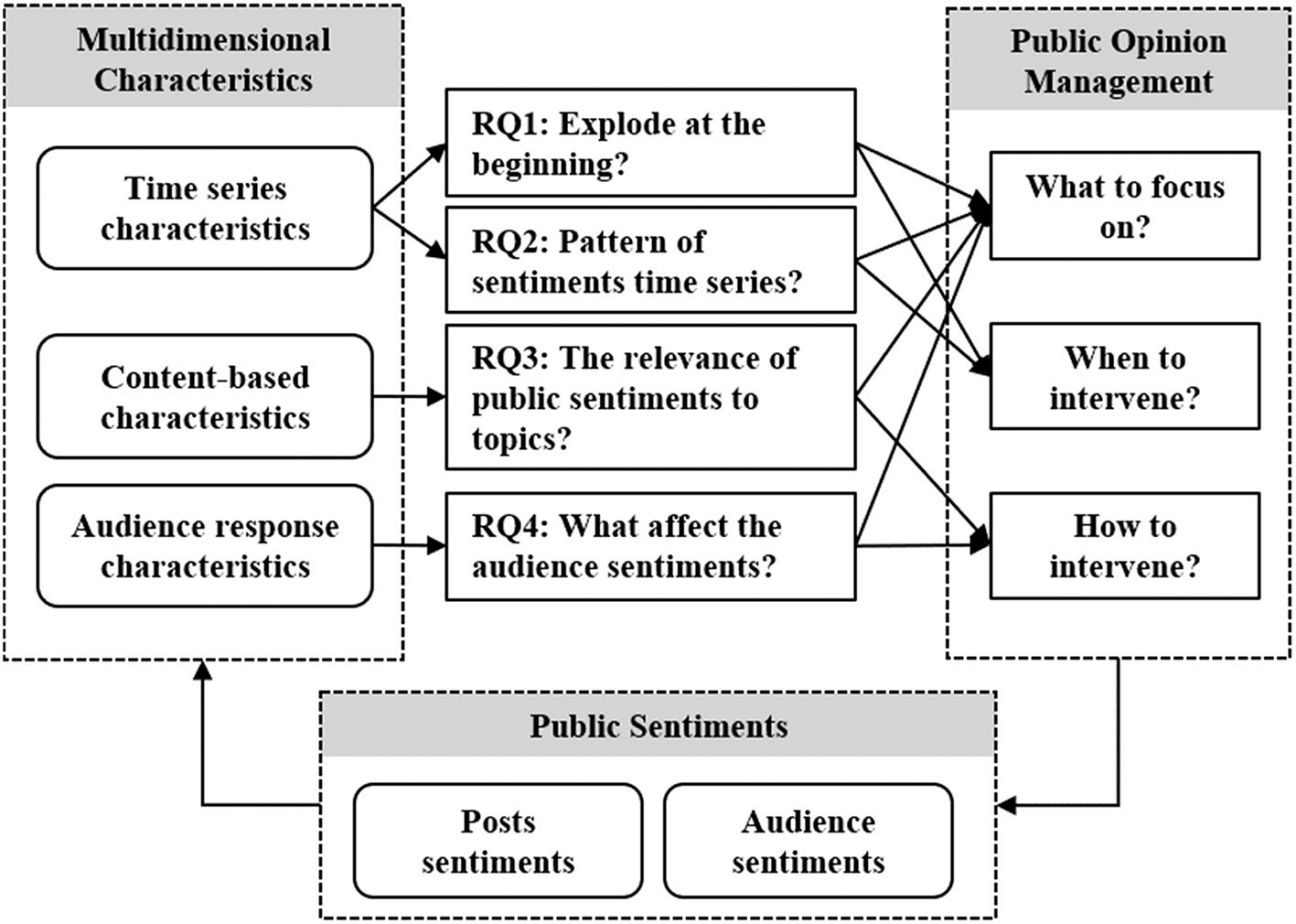
The multidimensional characteristics of public sentiments in public opinions management during the public emergencies.

The investigation of the above four questions can serve as the prerequisites for public opinion management as shown in Figure 1. Besides the accurate analysis combined with public sentiments and public discussion topics, this study should further mine and quantify the time series characteristics, content-based characteristics, and audience response characteristics of public sentiments. Accordingly, we obtained evidence-based insights and a scientific basis for public opinion management during public emergencies.

## 3. Methods

### 3.1. Events selection and data collection

Weibo is the leading online social network in China. Our data sources were mainly Weibo data provided by the “Zhiwei Data Sharing Platform” (http://university.zhiweidata.com/). Specifically, we selected two public opinion events including “The Wuhan Red Cross” during the outbreak period and “Fangfang Diary” in the stable period of the pandemic [newly confirmed domestic cases in mainland China that had declined to the single digits (50)]. This study assessed the manifestations of public sentiments properties by mining and analyzing the public participation behaviors on social media of these two public opinion events during COVID-19. Detailed information of the two events data was provided in Table 1 after the noise removal process.

**Table 1:** Details of the two events and their proportions of different sentiments.

**Events name**	**The Wuhan Red Cross**	**Fangfang Diary**
Events details	The use of epidemic supplies by the Wuhan Red Cross was questioned by the public, and the subsequent investigation sparked more public opinion and issues	The serialized content of Fangfang diary was questioned and continued to be discussed by netizens after its publication
Total in Weibo posts/comments	21,849/1,251,121	51,755/560,582
Tag 0 in Weibo posts/comments	12.5/9%	1.7/4.8%
Tag 1 in Weibo posts/comments	59.4/45.6%	74.2/50.8%
Tag 2 in Weibo posts/comments	24.1/39.8%	20.3/40.9%
Tag 3 in Weibo posts/comments	4/5.6%	3.8/3.5%

### 3.2. Computation and portrayal of public sentiment changes

Although people's language expressions on social networks are complex, we can perform a sentiment analysis on public-comments interaction data to determine their real attitudes. The experimental steps were as follows: First, Weibo and comments data of the selected events were obtained. We found that there were obviously many negative sentiments of the public from the event data, so we distinguished the public sentiments by the negative degree. Second, emotions are influenced by life experiences, public sentiments were classified according to the definition of basic emotions by the negative degree (56). Thus, the four levels label system was constructed: Tag 0 was the category without negative sentiments; Tag 1 was the slightly negative category, which mostly used sarcastic language and tended to be neutral; Tag 2 was the category with common negative sentiments such as sadness; Tag 3 was the category with extreme negative sentiments such as extreme anger and hysteria. A total sample size of *n* = 80,000 data set was constructed by manually marking data with this label system.

Third, the training set could be trained to form high-accuracy classification model based on the pretraining models (57, 58). The pretraining models were conducted fine tuning on the basis of the four levels label system to adapt the models to the language environment of this study. Then the performance of three pretraining models was tested to determine the unique pretraining model. We plotted the classification results as a time distribution curve for each sentiment classification to accurately determine the change in expressed emotions. The metrics of three commonly used pretraining models in various aspects were shown in Table 2. All three models were tested with a training set (validation set = 19:1) and a total sample size of *n* = 80,000. Although BERT-rbt3 was the fastest due to its compact size and light weight, Roberta had better performance in terms of accuracy, recall, f1_score, and precision. Therefore, using Roberta for sentiment calculation resulted in a more accurate description of public sentiments. The sentiment classification results were presented in Table 1.

**Table 2:** Comparison of the performance of different pretraining models.

**Model**	**BERT-base (slow)**	**BERT-rbt3 (fast)**	**Roberta (slow)**
Accuracy	0.8521624	0.8521224	0.8874552
Loss	0.4466113	0.41861752	0.39843366
Recall	0.8437177	0.84183085	0.8628877
F1_score	0.84751683	0.84482026	0.8602965
Precision	0.85252285	0.849068	0.8674692

### 3.3. Public opinion extraction and the relevance of public sentiments to topics

To explore the real and implicit opinions, the themes of people's expressed contents based on the results of sentiment classification were extracted. Public opinions were analyzed based on the LDA (latent Dirichlet allocation) model (59).

**Figure 2:**
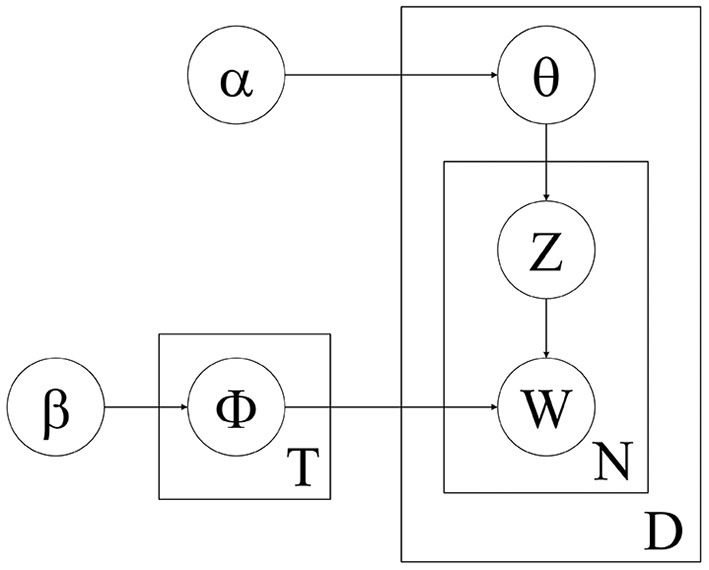
Topic generation principle of the LDA model.

The LDA model is an unsupervised method based on the bag of words assumption: Select a topic T with a prior probability distribution Φ that follows Dirichlet distribution β, and then select a word W in topic T and also in bag of words Z with a prior probability distribution θ that follows Dirichlet distribution α. The above steps are repeated until the entire article was completed. This process was shown in Figure 2. The process of using the LDA model is the reverse of the above process. The keywords of each topic were extracted after the predefined classification topics to analyze the content expressed by the public to obtain the information of people's real implicit opinions.

Using thematic clustering analysis to mine the contents of public expressions, specific topics in the public agenda were accurately excavated. Several public discussion issues of the two events were finally clustered into multiple topics by using the LDA topic clustering model, and the details of the topics were shown in Table 3.

**Table 3:** Clustered topics and details of two events.

**Events names**	**Topic ID**	**Topic details**
The Wuhan Red Cross	Topic 1	There were rumors that the Wuhan Red Cross sold 350 tons of vegetables donated by Shouguang.
	Topic 2	The Wuhan Red Cross responded to the sale of vegetables donated by Shouguang.
	Topic 3	Wuhan Commerce Bureau admitted to selling vegetables donated by Shouguang at low prices.
	Topic 4	Wuhan Red Cross's use of donated materials were questioned by public.
	Topic 5	China Red Cross Federation sent a working group to Wuhan.
	Topic 6	China Red Cross Federation demanded thorough rectification in Hubei.
	Topic 7	Hubei Red Cross severely punished those relevant responsible.
	Topic 8	Reporters secretly visited the Wuhan Red Cross warehouse.
	Topic 9	Private enterprises took over the placement of donated materials from the Wuhan Red Cross.
Fangfang Diary	Topic 1	Fangfang Diary's serialized content led to heated debate.
	Topic 2	Fangfang Diary described her niece's departure from Wuhan.
	Topic 3	Fangfang's small property right villa turned positive and made a profit of 10 million.
	Topic 4	Fangfang Diary spread rumors about Nurse Liang's death.
	Topic 5	Fangfang Diary was widely published overseas.
	Topic 6	Fangfang Diary aroused controversy.
	Topic 7	Fangfang asked academician Zhang Boli to apologize.
	Topic 8	Fangfang's friend Liang Yanping was punished and Fangfang was again controversial.

After keywords coding of the theme analysis results, the theme content and public sentiment were cross analyzed by using KHcoder (http://khcoder.net/en/). Based on the cross-analysis results of text topics coding combined with sentiments calculation, the relationships between different sentiments negative levels and text topics were obtained, so as to understand the relevance between public sentiments and topics discussed by the public.

Public sentiments were classified into different levels by using the preceding pretraining model in all the texts of Weibo posts and Weibo comments. The cross-analysis results showed the proportion of texts for each topic in each sentiment category. Since multiple topics could appear in one text, the sum of all topics in the text might exceed 100%. In order to understand the relevance between public sentiments and public discussion topics, that is, the characteristics of public sentiments in public discussion content. The impact of public sentiments on public topics needed to be quantified.

Texts of each sentiment category had different quantities, and the proportion of each topic in all texts was also different. Thus, it was necessary to measure the impact of public sentiments on the proportion of public topics in texts in a standardized way. If the proportion of texts for Topic (j) in texts for the Tag (i) public sentiments was P_ij_, and the percentage variation of P_ij_ relative to the proportion of texts for Topic (j) in all texts P_j_ was V_ij_, then


$$\begin{array}{l} V_{ij}={\left(\frac{\left(P_{ij}-P_{j}\right)}{P_{j}}\right)}^{*}100\% \end{array}$$

This indicator was used to quantify the impact of public sentiments at different levels on public topics, and the quantitative analysis results of content-based characteristics were obtained.

### 3.4. Correlation analysis of audience sentiments combined with complex networks

Besides time series characteristics and content-based characteristics, it was also significant to pay attention to audience response characteristics. Audience response characteristics reflected by audience sentiments determined what interventions were more effective. As a consequence, this study analyzed the relevant characteristics of audience sentiments for each Weibo post and its corresponding Weibo comments, and established relationships between posts and their audience. After excluding irrelevant variables such as user ID, URL, Weibo post ID, and combining similar variables, the correlation analysis was performed on the remaining variables to determine the characteristics of audience sentiments. What needed further explanation was that there were many missing values of the User's geographic location variable, and the authenticity of the location information of social media users could not be verified, so the correlation of this variable could also not be verified. Hence, this variable was discarded in this study. The final remaining 12 variables were shown in Table 4.

**Table 4:** Variable names and variable meanings.

**ID**	**Variable name**	**Variable meaning**
1	WPS	Sentiment classification of each Weibo post
2	WPT	The length of time each Weibo post was posted
3	WPC	The total number of comments for each Weibo post
4	CP0	Proportion of comments with Tag 0 for each Weibo post
5	CP1	Proportion of comments with Tag 1 for each Weibo post
6	CP2	Proportion of comments with Tag 2 for each Weibo post
7	CP3	Proportion of comments with Tag 3 for each Weibo post
8	WPL	The number of likes for each Weibo post
9	WUF	The number of followers of the user who posted Weibo
10	WUT	Authentication type of the user who posted Weibo
11	WUP	The number of Weibo posts of the user
12	WUG	The gender of the user

There were three category variables in the 12 variables, for the sake of ensuring the robustness of correlation analysis, Spearman correlation coefficient and Pearson correlation coefficient were both used for correlation investigation. Finally, we embedded the sentiment calculation results of Weibo posts and comments into the corresponding retweet relationships to form an emotion network. Following the principle of complex networks, Gephi was used to map the emotion network to discover gathering and forwarding of various sentiments. Then the relationship between public sentiments and other factors were portrayed.

## 4. Results

### 4.1. Patterns of public sentiments in time series

Given the public emotion's diverse and complex nature, there were trends of public sentiments and some implicit emotional needed to be identified. According to the life cycle theory, the event development trend can be divided into three stages: initial, outbreak, and ending. The data on the timeframe of the event outbreak indicated that although each event lasted for different lengths of time and had different levels of popularity, they all had similar trends.

**Figure 3:**
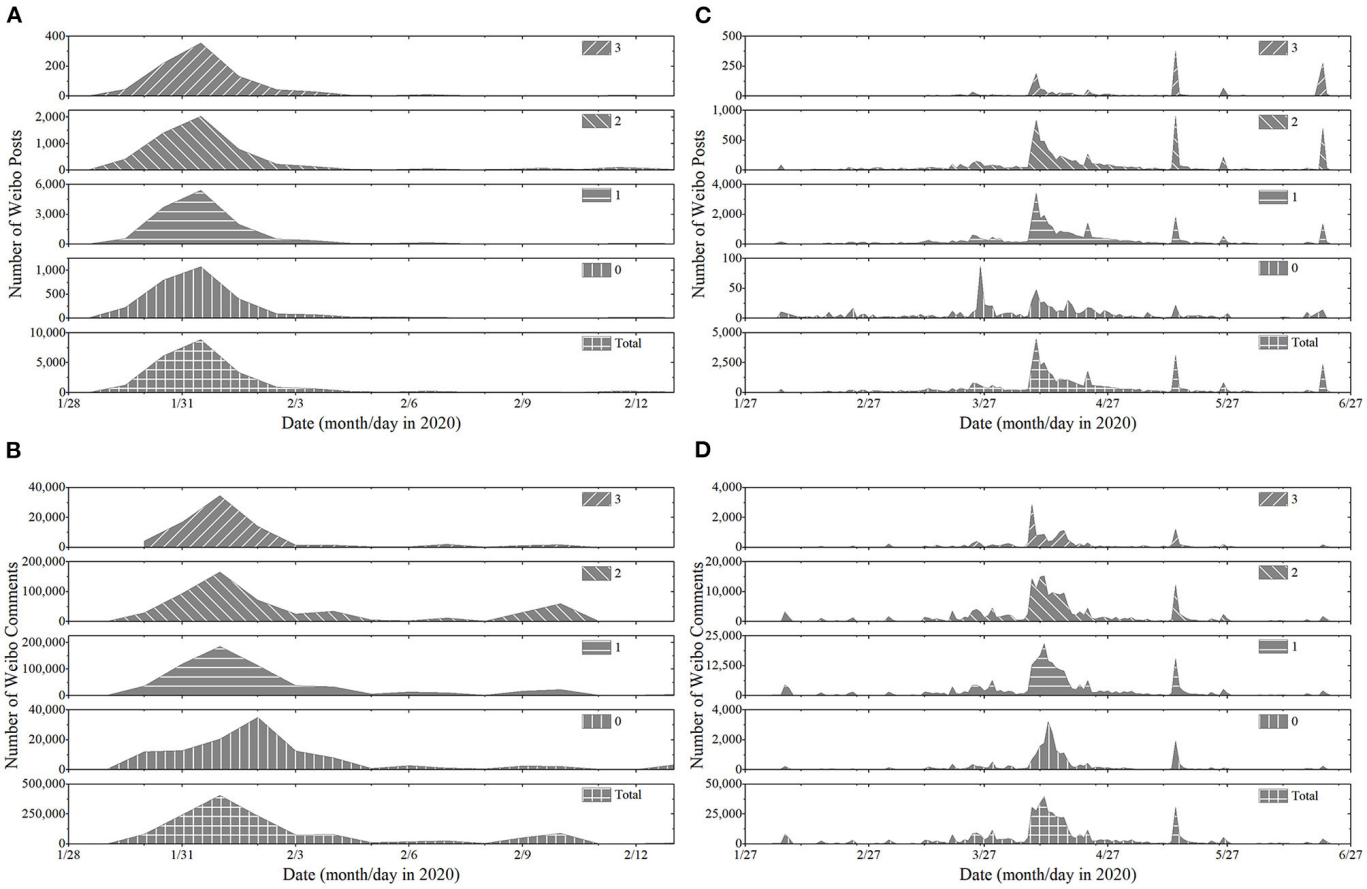
The time series of different public sentiments of “The Wuhan Red Cross” and “Fangfang Diary”. **(A)** Posts sentiments of “The Wuhan Red Cross”. **(B)** Audience sentiments of “The Wuhan Red Cross”. **(C)** Posts sentiments of “Fangfang Diary”. **(D)** Audience sentiments of “Fangfang Diary”.

As shown in Figure 3, “The Wuhan Red Cross” and “Fangfang Diary” had high public discussion popularity. In particular, “The Wuhan Red Cross” was the event during the outbreak of the pandemic, and its related topics were discussed in a relatively concentrated time. A number of issues had emerged in succession, and public sentiments had rapidly developed explosively after a short period of priming. “Fangfang Diary” started in the outbreak period of the epidemic and broke out in the stable period of the pandemic due to a key issue. The public sentiments had a long period of priming, so there were multiple peaks within the event. Although the duration of the two events was inconsistent, the consensus was that public sentiments had a foreshadowing period and did not erupt at the beginning. This conformed to the interpretation of the life cycle theory and agenda setting theory, and also answered the RQ1.

In particular, in “The Wuhan Red Cross” and “Fangfang Diary”, the peak of audience sentiments came later than that of posts sentiments, except for three exceptions: First, the peak lag of the audience sentiments of Tag 0 in “The Wuhan Red Cross” were more obvious in Figure 3B. This phenomenon was due to the emergence of topics about the government's response measures to the event. The emergence of some recognized public sentiments had significantly delayed the real peak of the non-negative sentiments represented by Tag 0. Second, in the “Fangfang Diary” as shown in Figure 3C, the peak of Tag 0 posts sentiments was significantly earlier than that of other level sentiments, and the subsequent amount of Tag 0 posts sentiments was less. In the free discussion of public agenda, there was no content about the official release of deterministic information by the government. At the same time, the negative increase of public sentiments showed the importance of official deterministic information compared with “The Wuhan Red Cross”. Third, in the Figures 3C, D, the peak value of Tag 3 public sentiments appeared at different times in posts sentiments and audience sentiments. This phenomenon reflected the uncertainty of extremely negative public sentiments.

**Figure 4:**
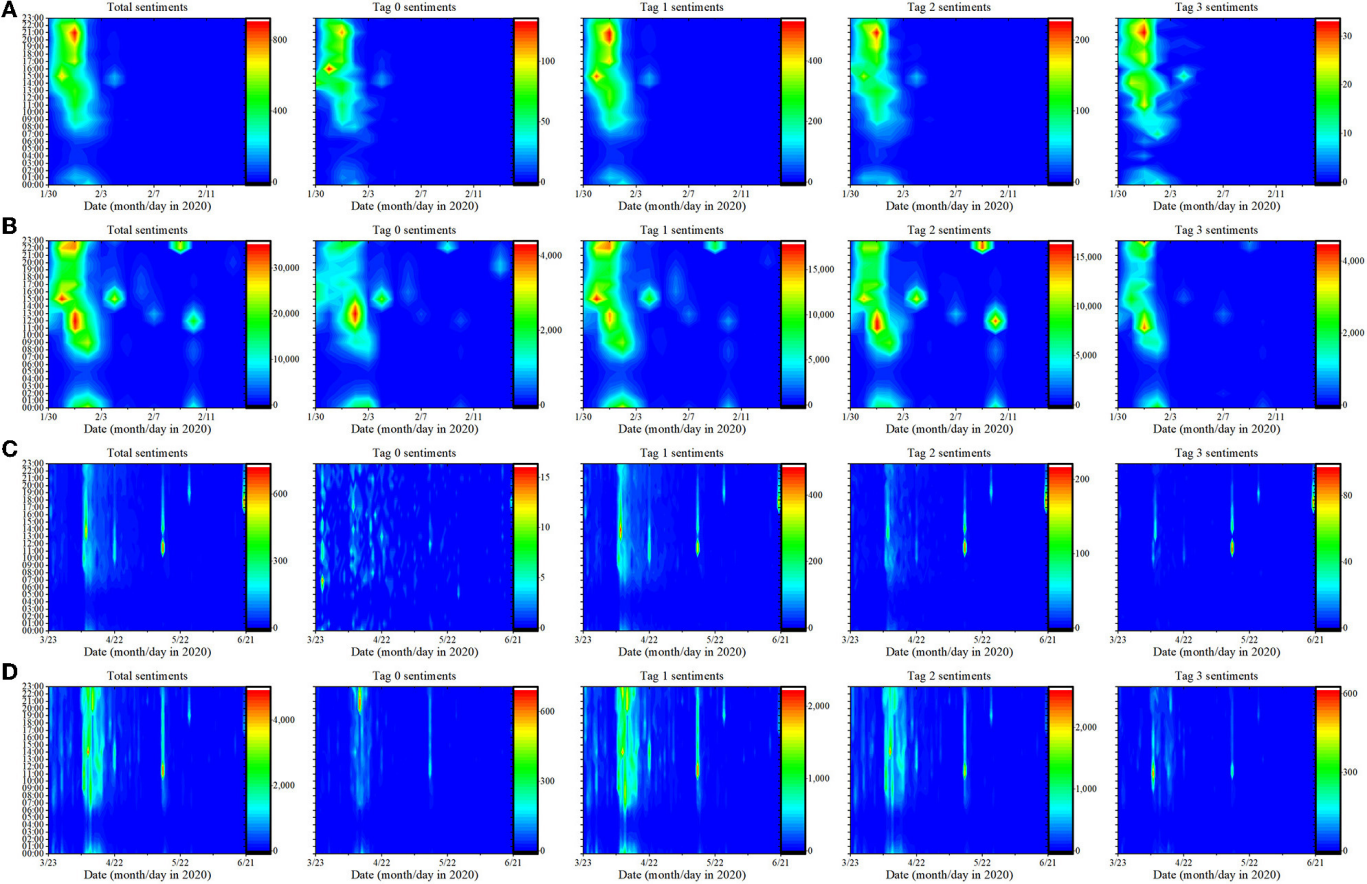
The time series of different public sentiments in 24h of “The Wuhan Red Cross” and “Fangfang Diary”. **(A)** Posts sentiments of “The Wuhan Red Cross”. **(B)** Audience sentiments of “The Wuhan Red Cross”. **(C)** Posts sentiments of “Fangfang Diary”. **(D)** Audience sentiments of “Fangfang Diary”.

When the time series of public sentiments were presented in a 24-h cycle, the new pattern was displayed, as shown in Figure 4. On the whole, the trend of public sentiments time series was consistent with that in Figure 3. Although the communication patterns of time series for different public sentiments were somewhat different, the peak of public sentiments occurred at noon and 2 h before midnight. The gathering of public sentiments at noon and before midnight indicated that the emergence pattern of public sentiments was closely related to the public's work and rest time. At noon and before midnight, public were in a state where it was easier to focus on expressing emotions and participating in discussion of issues. Public attention had shifted from work and life to public agendas.

The appearance of public sentiments had obvious gap, and the gap on the trend of public sentiments can be used as the window period for controlling public sentiments. Most people were unable to participate in the public discussion during this window period. Therefore, using this window period was a significant aspect of public sentiments management. Combined with Figures 3, 4, it can be found that the peak of Tag 3 audience sentiments was more concentrated in the outbreak stage of public sentiments, and the peak of Tag 3 posts sentiments still appeared obviously in the later period of the event. Although the duration of “The Wuhan Red Cross” and “Fangfang Diary” events was different, the audience sentiments all showed the weakening of extreme negative sentiments. The uncertainty of extreme negative public sentiments was clearly displayed. The above phenomenon revealed the communication patterns of public sentiments mentioned in RQ2.

Changes in public sentiments revealed communication patterns of public sentiments time series. Based on the above characteristics of the public sentiments time series, we can select a more accurate time window to implement interventions and prevent public sentiments from becoming harmful.

### 4.2. The relevance of public discussion topics and public sentiments

**Figure 5:**
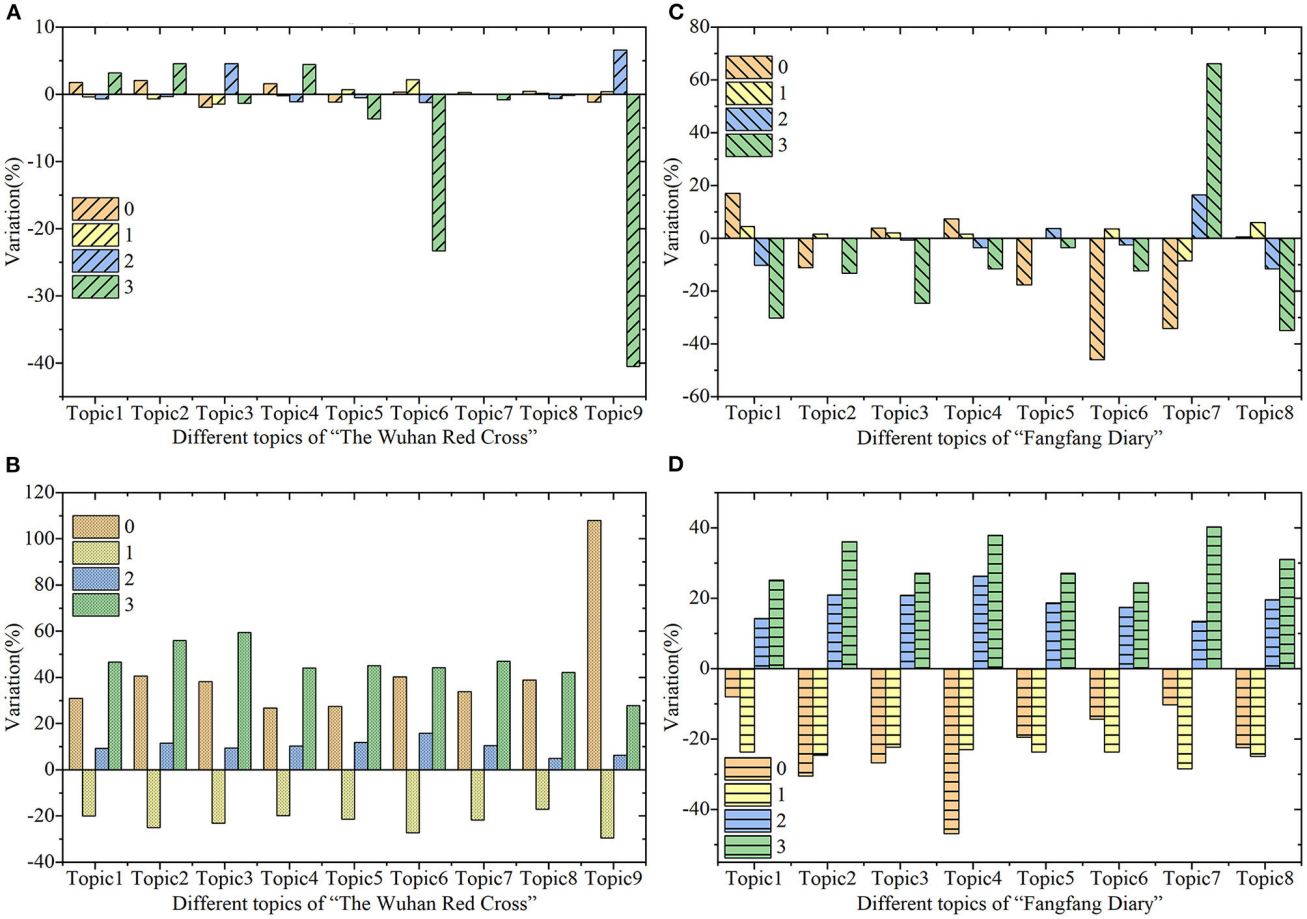
The percentage variation of the topics content proportion, difference of four levels public sentiments in different topics. **(A)** The variation of the Weibo posts in “The Wuhan Red Cross”. **(B)** The variation of the Weibo comments in “The Wuhan Red Cross”. **(C)** The variation of the Weibo posts in “Fangfang Diary”. **(D)** The variation of the Weibo comments in “Fangfang Diary”.

In “The Wuhan Red Cross” and “Fangfang Diary”, the content-based characteristics of posts sentiments and audience sentiments were analyzed combined with topics features, as shown in Figure 5.

From the relevance analysis of public discussion topics and public sentiments, three content-based characteristics of public sentiments were found: First, as shown in Figures 5A, C, there was no obvious relevance between posts sentiments and public discussion topics in Weibo posts. Public sentiments had no impact on the public discussion content in Weibo posts, while the audience sentiments in Weibo comments had a more patterning impact, as shown in Figures 5B, D. This phenomenon showed that the audience sentiments of Weibo posts had an impact on Weibo comments under certain patterns. Second, as shown in Figures 5B, D, the pattern of this impact was that the more negative the audience sentiments were, the more texts related to the public discussion topics were in Weibo comments. This indicated that the more negative the public sentiments were, the higher the discussion and participation of audience were in the public agenda. The Weibo comments released by people with more negative emotions were more relevant to public discussion topics. Third, although the audience sentiments of Tag 0 in Figures 5B, D played opposite roles in different events, they had the same impact on the same public agenda. However, the Weibo comments published by the audience sentiments of Tag 1 were not closely related to the public discussion topics, and their expressions contained much useless information. The above three content-based characteristics of public sentiments were the answers to RQ3. Consequently, in addition to paying attention to the more negative public sentiments, we should also pay attention to what people who do not have negative public sentiments discuss.

### 4.3. The correlation analysis of audience sentiments

The previous experimental results illustrated the content-based characteristics of audience sentiments. Which facts were related to audience sentiments? Did those who published Weibo posts have an impact on audience sentiments? Here we analyzed the correlation of 12 variables to explore the answer to RQ4. The research results of RQ4 reflected the audience response characteristics.

The correlation analysis results for “The Wuhan Red Cross” were shown in Tables 5, 6, and the correlation analysis results for event “Fangfang Diary” were shown in Tables 7, 8. The four variables CP0, CP1, CP2, and CP3 represented the standardized audience sentiments. The correlation analysis results between them and other variables showed the following phenomena of audience sentiments: First of all, the audience sentiments in the two events were independent of the corresponding post sentiments. Second, combining the results of the two events, the audience sentiments were independent of the length of time that the Weibo posts had been published and the comments and likes of the Weibo posts. Third, the number of followers for users of a certain Weibo post and the number of Weibo posts published by the user had no obvious relationship with the audience sentiments. However, there were different results on the correlation between audience sentiments and user authentication types and genders. In the event “The Wuhan Red Cross” during the outbreak of the pandemic, there was a clear correlation between the audience sentiments of Tag 0 and the user authentication type and gender, and the audience sentiments of Tag 1 were related to the gender of the user. In the event “Fangfang Diary” in the stable period of the pandemic, there was no significant correlation between audience sentiments and user authentication type and gender.

**Table 5:** Spearman correlation coefficient of the event “The Wuhan Red Cross”.

	**WPS**	**WPT**	**WPC**	**CP0**	**CP1**	**CP2**	**CP3**	**WPL**	**WUF**	**WUT**	**WUP**	**WUG**
WPS	1											
WPT	0.072^**^	1										
WPC	0.035	0.016	1									
CP0	−0.058	0.127^**^	−0.194^**^	1								
CP1	0.018	0.113^**^	−0.031	−0.114^**^	1							
CP2	0.025	−0.158^**^	0.087^**^	−0.327^**^	−0.653^**^	1						
CP3	−0.011	−0.146^**^	−0.344^**^	−0.114^**^	−0.196^**^	0.128^**^	1					
WPL	−0.021	−0.026	0.068^*^	−0.023	0.080^**^	−0.043	−0.055^*^	1				
WUF	−0.026	−0.002	0.246^**^	0.072^**^	−0.051	0.084^**^	−0.081^**^	−0.011	1			
WUT	−0.025	−0.103^**^	−0.006	−0.118^**^	−0.007	−0.063	−0.028	0.029	−0.384^**^	1		
WUP	0.010	0.017	0.012	0.134^**^	−0.006	0.054^*^	0.026	0.144^**^	0.397^**^	−0.374^**^	1	
WUG	−0.025	0.004	0.020	0.100^**^	0.117^**^	−0.064	−0.017	−0.074^**^	0.071^**^	−0.097^**^	0.078^**^	1

** Correlation is significant at the 0.01 level (2-tailed);

* Correlation is significant at the 0.05 level (2-tailed).

**Table 6:** Pearson correlation coefficient of the event “The Wuhan Red Cross”.

	**WPT**	**WPC**	**CP0**	**CP1**	**CP2**	**CP3**	**WPL**	**WUF**	**WUP**
WPT	1								
WPC	0.118^**^	1							
CP0	0.027	−0.025	1						
CP1	0.046	−0.060^*^	−0.315^**^	1					
CP2	−0.040	0.083^*^	−0.338^**^	−0.681^**^	1				
CP3	−0.058^*^	−0.003	−0.101^**^	−0.310^**^	−0.078^**^	1			
WPL	0.055^*^	0.125^**^	0.021	0.016	−0.024	−0.016	1		
WUF	0.159^**^	0.541^**^	−0.004	−0.010	0.044	−0.055	0.035	1	
WUP	0.033	0.145^**^	−0.017	0.033	−0.003	−0.042	0.020	0.425^**^	1

** Correlation is significant at the 0.01 level (2-tailed);

* Correlation is significant at the 0.05 level (2-tailed).

**Table 7:** Spearman correlation coefficient of the event “Fangfang Diary”.

	**WPS**	**WPT**	**WPC**	**CP0**	**CP1**	**CP2**	**CP3**	**WPL**	**WUF**	**WUT**	**WUP**	**WUG**
WPS	1											
WPT	−0.043	1										
WPC	0.190^**^	−0.109^**^	1									
CP0	−0.041	0.102^**^	−0.081^*^	1								
CP1	−0.124^**^	0.012	0.017	0.176^**^	1							
CP2	0.110^**^	−0.036	0.023	−0.389^**^	−0.912^**^	1						
CP3	0.110^**^	0.047	−0.052	−0.181^**^	−0.385^**^	0.237^**^	1					
WPL	0.182^**^	−0.109^**^	0.854^**^	−0.019	0.075^*^	−0.050	0.014	1				
WUF	−0.028	−0.034	0.342^**^	−0.020	0.152^**^	−0.114^**^	−0.048	0.416^**^	1			
WUT	−0.004	0.033	−0.176^**^	−0.060	−0.090^*^	0.076^*^	−0.023	−0.228^**^	−0.401^**^	1		
WUP	−0.072	0.027	0.133^**^	−0.140^**^	0.076^*^	−0.027	0.027	0.163^**^	0.615^**^	−0.312^**^	1	
WUG	−0.051	0.077^*^	−0.050	−0.054	−0.036	0.052	0.000	−0.143^**^	0.005	−0.214^**^	0.189^**^	1

** Correlation is significant at the 0.01 level (2-tailed);

* Correlation is significant at the 0.05 level (2-tailed).

**Table 8:** Pearson correlation coefficient of the event “Fangfang Diary”.

	**WPT**	**WPC**	**CP0**	**CP1**	**CP2**	**CP3**	**WPL**	**WUF**	**WUP**
WPT	1								
WPC	−0.087^*^	1							
CP0	0.118^**^	0.049	1						
CP1	−0.015	−0.042	0.076^*^	1					
CP2	−0.035	0.012	−0.398^**^	−0.909^**^	1				
CP3	0.024	0.041	−0.137^**^	−0.338^**^	0.107^**^	1			
WPL	−0.030	0.694^**^	0.031	−0.044	0.026	0.020	1		
WUF	−0.030	0.249^**^	−0.057	0.007	0.021	−0.028	0.310^**^	1	
WUP	0.060	−0.003	−0.121^**^	0.001	0.034	0.030	0.035	0.501^**^	1

** Correlation is significant at the 0.01 level (2-tailed);

* Correlation is significant at the 0.05 level (2-tailed).

**Figure 6:**
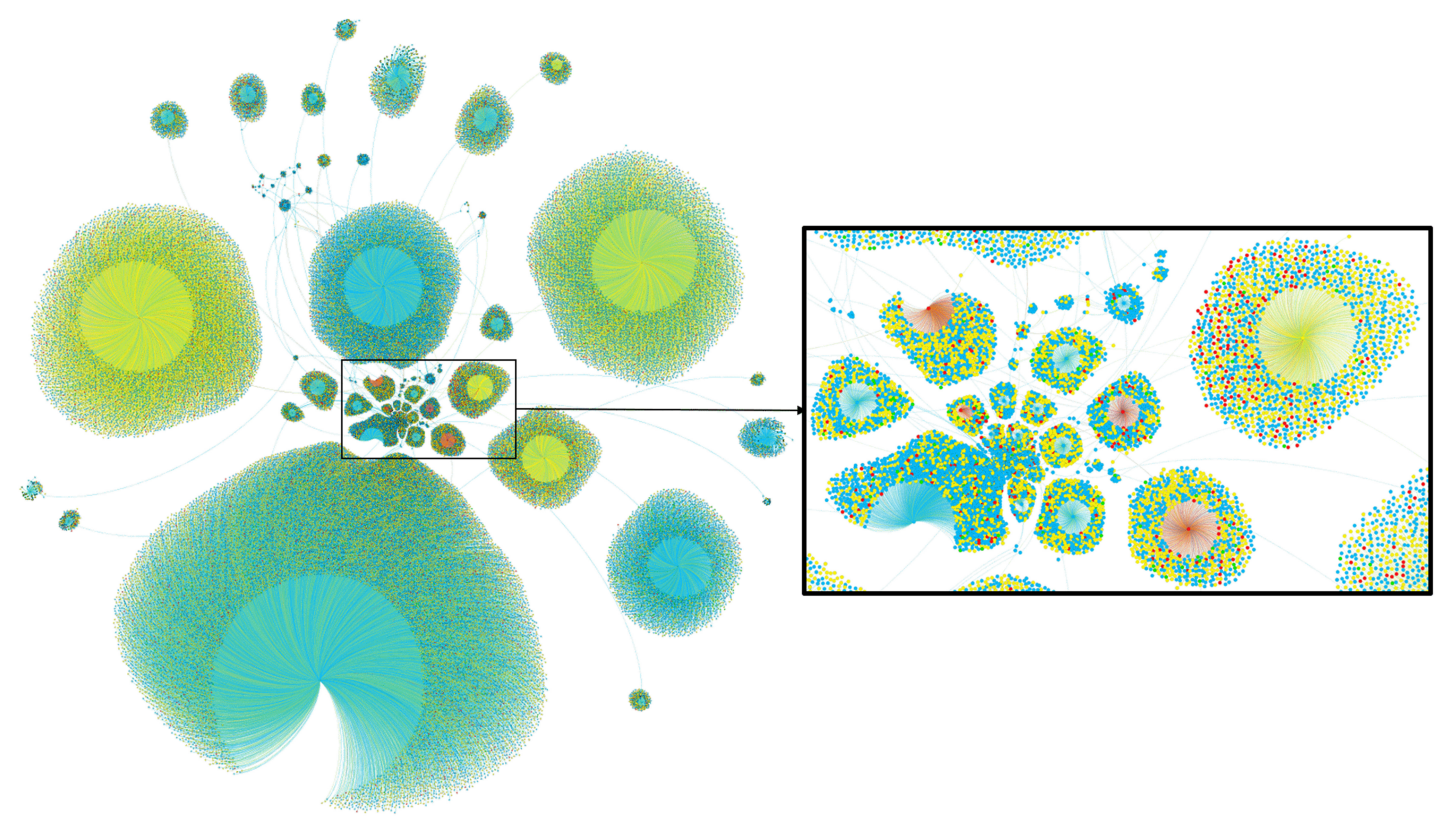
Part of the sentiments forwarding network in Weibo posts and comments (green: Tag 0; blue: Tag 1; yellow: Tag 2; red: Tag 3).

The above phenomena indicated two audience response characteristics shown by audience sentiments: First, the audience sentiments had no obvious relationship with the corresponding Weibo post and the published user. Secondly, the special point was that under the premise of structural tension such as the pandemic, the low negative level of audience sentiments had a certain correlation with user authentication types and gender. The spread of negative audience sentiments was not affected by these factors. As shown in Figure 6 and the previous results, public discussion and public sentiments were only clustered around opinion leaders. Opinion leaders cannot affect public sentiments. The steering role of opinion leaders had been weakened in recent social network environment. As a consequence, the answer to RQ4 indicated that the right person should be selected for guidance when considering the implementation of intervention.

## 5. Discussion

Whether in the pandemic outbreak or in the current period of normalized management, the emergence of public opinion had posed a double challenge to the actual epidemic management and online public opinion management. The management and intervention of public sentiments also became the most prominent contradiction in public opinion management. The correct information needed to be smoothly disseminated to the public and the problem of public sentiments needed to be solved first. The exploration of multidimensional public sentiments characteristics not only played a great role in mitigating the impact of public long-term psychological issues, but also had a practical significance for the public opinion management in social network. The analysis of the multidimensional public sentiments characteristics in this study can offer support for public opinion management in several aspects, include time series, content, and audience. This study also provided suggestions for managing public sentiments caused by public opinion in social media during the pandemic.

### 5.1. Insights and significance on emergency management in public opinion communication

Managers need to consistently monitor public sentiments during public opinion. Attention should be paid to multidimensional characteristics of public sentiments. First, find out the window period of intervention from the time series characteristics. This study found increased negativity in public sentiments during the pandemic, and also emphasized that the outbreak of public sentiments was not at the initial stage of public discussion of relevant issues. According to the interactive ritual chain theory, public opinion events bring people together to discuss relevant topics as the public tries to reduce its concerns, and receive information, and emotional support through communication, from which the social atmosphere in public opinion events is then formed (28). Thus, the public sentiments got the priming period. For the more negative the public sentiments were, the higher the uncertainty was. This period was the first critical point of public sentiments management and it's not easy to catch. The second critical point of public sentiments management was to select an appropriate window period according to the gap of the public sentiments time series pattern specified in this study.

Second, considering the difference between posts sentiments and audience sentiments, the specific intervention strategy and information were determined by combining the content-based characteristics of public sentiments. Since audience sentiments were more modular, and the more negative the audience sentiments were, the more relevant the discussion content was to public discussion topics. It illustrated that people as audience were more likely to be affected by events to produce more negative sentiments. However, their more in-depth participation in the discussion showed that they were more concerned about the development of events and relevant information. Therefore, combined with the differences in government intervention in “The Wuhan Red Cross” and “Fangfang Diary”, the official release of certainty information might have a positive effect on the management of audience sentiments, which needed further verification by subsequent research. Such discussions and audience sentiments undoubtedly aggravated the spread of public opinion, and the spread of misinformation or disinformation at the same time. It also demonstrated that the management of public sentiments was crucial.

Third, the characteristics of audience sentiments also play a role in suggesting the implementation of specific interventions. Audience sentiments were irrelevant to Weibo posts and users, which also told us that in social network at the moment, public sentiments would not be guided because the other party had a large number of fans or the other party was an opinion leader. The formation of emotion was related to the public cognition of public opinion events, which was related to individual differences and environmental factors. The role of opinion leaders in social media had been changed. Opinion leaders could only influence what topics people discussed, not the public opinion. This is consistent with the views of agenda setting theory. In the context of the pandemic, public was in a state of tension, and non-negative audience sentiments changed because of the authentication type and gender of users. It was worth noting that although there was overlap between data and topics, the conclusions on gender impact were different from previous studies (48). Not only the user gender was irrelevant to Weibo posts, but the correlation between audience sentiments and gender was also different from the conclusions of previous studies. Therefore, the management of public sentiments needed to fully consider the audience response characteristics shown by audience sentiments, and the ideal intervention measures needed to be cautiously considered.

For the sake of social stability, we need to be deeply aware of the importance of effective and timely intervention in emotional guidance and public sentiments management. In view of the increase in emotional issues caused by the increase in people's use of social media during the COVID-19 pandemic (38), this study provided a method to understand some emotional problems. This research also provided a method for measuring changes in the public sentiments and quantitatively described the multidimensional characteristics of public sentiments. Therefore, the practical significance of this study was that it provided a scientific basis for managers to develop effective strategies for public sentiments management. It also enabled this research to provide certain requirement for public opinion management in terms of public sentiments.

### 5.2. Limitations and future research

To maximize the practical value of this study, we must avoid the effects of several limitations. First, the integrity of the data obtained from Weibo was difficult to guarantee and there was some interference in the complete portrayal of sentiment trends. The duration of Weibo information was restricted and posts were not available after subsequent deletion; therefore, sample completeness was problematic. Due to the privacy protection policy, we cannot confirm the gender and geographic information of users, and the authenticity of the users' reported gender and location was uncertain. Therefore, the related characteristics cannot be accurately reported.

Second, our data were limited to the domestic Weibo section. We did not find effective access to relevant social media data abroad; therefore, public perceptions of some of the events were limited to the domestic context, which compromised the comprehensiveness of the problem description and interpretation. Moreover, there was a small amount of error in the results of classification using pretraining model. Although the method used has been the most efficient and accurate method for processing massive data classification, the existence of biased results cannot be avoided for the time being.

Finally, although Weibo is a widely used social media platform, many members of the public (for example, the elderly who cannot use the Internet) do not disclose their thoughts on any platform. There were some individuals chose to view social media without interacting who were similarly infected by public emotions. We were unable to identify this population from the social media data or address other issues that might exist.

At present, research on public sentiments on short video platforms like TikTok have emerged (60), but investigations focused on different platforms were not enough. Future research should analyze how people communicate their emotions on social media and explore the motives and inner rules of the behaviors on multiple platform so as to explore the mechanism of improving public opinion management and more accurately support the maintenance of it.

## 6. Conclusion

This research expanded the empirical evidence to explore multidimensional attributes and characteristics of public sentiments during the COVID-19 pandemic. The formulation of effective intervention strategies required the government to consider the time series characteristics, content-based characteristics, and audience response characteristics of public sentiments in public opinion management. The study found that the time series characteristics of public sentiments reminded of the trends of public sentiments at different negative levels and the existence of window periods. Content-based characteristics of public sentiments indicated that the more negative the audience sentiments were, the more relevant they were to public discussion topics. Such people had higher participation in the discussion and deeper participation behavior. Audience response characteristics of public sentiments proved that the emergence of audience sentiments was independent from Weibo posts and users, and the steering role of the opinion leaders produced no noticeable effect in changing audience sentiments. Based on the above three characteristics, the management and intervention strategy for public sentiments in public opinion communication can generate more effective positive effects. The above research results will also provide some support for improving the ability to respond public opinion and enhancing the system of public opinion management.

## Data availability statement

The original contributions presented in the study are included in the article/supplementary material, further inquiries can be directed to the corresponding author.

## Author contributions

NM and GY conceived and designed this study. NM collected and interpreted the data, drafted the manuscript, and with guidance from GY revised the manuscript for important intellectual content. NM, XJ, and XZ solved the use of the algorithm and the visualization of the results. All of the authors reviewed, discussed, and approved the final manuscript.
